# MicroRNA-133a impairs perfusion recovery after hindlimb ischemia in diabetic mice

**DOI:** 10.1042/BSR20180346

**Published:** 2018-07-03

**Authors:** Lingdan Chen, Chunli Liu, Dejun Sun, Tao Wang, Li Zhao, Wenli Chen, Mingjie Yuan, Jian Wang, Wenju Lu

**Affiliations:** 1 State Key Laboratory of Respiratory Diseases, Guangzhou Institute of Respiratory Health, The First Affiliated Hospital, Guangzhou Medical University, Guangzhou, Guangdong, China; 2 Department of Respiratory Medicine, Inner Mongolia Autonomous Region People’s Hospital, Hohhot 010017, Inner Mongolia Autonomous Region, China; 3 Department of Cardiology, Renmin Hospital of Wuhan University, Wuhan 430071, China

**Keywords:** MicroRNA-133a, nitric oxide, angiogenesis, reactive oxygen species, peripheral arterial disease

## Abstract

**Objective:** Peripheral arterial disease (PAD) patients with diabetes mellitus suffer from impaired neovascularization after ischemia which results in poorer outcomes. MicroRNA (miR)-133a is excessively expressed in endothelial cells under diabetic conditions. Here, we test whether diabetes-induced miR-133a up-regulation is involved in the impaired capability of neovascularization in experimental PAD models. **Methods and results:** MiR-133a level was measured by quantitative RT-PCR and showed a higher expression level in the ischemic muscle from diabetic mice when compared with nondiabetic mice. Knockdown of miR-133a using antagomir improved perfusion recovery and angiogenesis in experimental PAD model with diabetes day 21 after HLI. On the other hand, overexpression of miR-133a impaired perfusion recovery. Ischemic muscle was harvested day 7 after experimental PAD for biochemical test, miR-133a antagonism resulted in reduced malondialdehyde, and it increased GTP cyclohydrolase 1 (GCH1), and cyclic guanine monophosphate (cGMP) levels. In cultured endothelial cells, miR-133a antagonism resulted in reduced reactive oxygen species level, and it increased tube formation, nitric oxide (NO), and cGMP level. Moreover, miR-133a antagonism-induced angiogenesis was abolished by GCH1 inhibitor. In contrary, miR-133a overexpression impairs angiogenesis and it reduces GCH1, NO, and cGMP levels in nondiabetic models. **Conclusion:** Diabetes mellitus-induced miR-133a up-regulation impairs angiogenesis in PAD by reducing NO synthesis in endothelial cells. MiR-133a antagonism improves postischemic angiogenesis.

## Introduction

Peripheral arterial disease (PAD) affects approximately 20% of the population over 55 years old worldwide [1–4], diabetes mellitus is an important risk factor for the development of PAD and puts PAD patients in a higher risk of lower extremity amputation and even death, which is mainly attributed to hyperglycemia-induced neovascularization impairment [1,2,5]. Ischemia-induced neovascularization is the key point of perfusion recovery for PAD; however, there is no-known medication that is able to induce enough functional blood vessel growth and thus treat PAD; especially when complicated with diabetes [6,7]. Mouse hindlimb ischemia (HLI) has been widely used as a preclinical PAD model, the processes involved in vascular adaptation following vessel occlusion are similar to PAD patients. Moreover, similar to human conditions, perfusion recovery after HLI in mice with diabetes is impaired, but the underlying mechanisms are not fully understood [8].

MicroRNAs (miRs) are a class of small noncoding RNA molecules containing approximately 18–25 nucleotides, which bind to the seed sequences in the 3′-untranslated region(3′-UTR) of mRNAs and induce mRNA degradation or suppress protein translation [9]. Recently, miRs have been reported to play important roles in tissue recovery and vascular remodeling after PAD [9]. MiR-133a, which has been found to be excessively expressed in the blood vessels under hyperglycemic condition, induces endothelial cell dysfunction through inhibition of endothelial nitric oxide synthase (eNOS) [10]. It is well known that among patients with diabetes, hyperglycemia decreases synthesis and increases degradation of nitric oxide (NO) [8,11]. Considering endogenous NO synthesis is essential for angiogenesis after tissue ischemia and miR133a is up-regulated under oxidative stress, the present study was designed to investigate the role of miR-133a in neovascularization after PAD and further elucidate the underlying mechanisms.

## Materials and methods

### Mice

Wild-type C57BL/6 J male mice (6 weeks old) were purchased from the Nanjing BioMedical Research Institute of Nanjing University (Nanjing, China). Animals were housed in the specific pathogen-free facilities, and the Animal Care and Use Committee of Guangzhou Medical University approved all experimental protocols. All methods were performed in accordance with the guidelines and regulations approved by the Animal Care and Use Committee of Guangzhou Medical University.

### Type 1 diabetes and hindlimb ischemia induction

Type 1 diabetes was induced by intraperitoneal injections of streptozotocin (STZ, Sangon Biotech, Shanghai, China). Male C57BL6 mice (8 weeks of age) were given multiple low-dose STZ (MLD-STZ), intraperitoneal doses of 40 mg/kg of STZ in citrate buffer (pH 4.5) for 5 days consecutively. The level of hyperglycemia in the experimental and control groups of mice were determined by measuring hemoglobin A1C (PTS Diagnostics, IN, U.S.A) and fast blood glucose as previously described [12], and the groups were found to have identical levels of hemoglobin A1C and fast blood glucose. Three weeks after diabetes induction, all nondiabetic and diabetic mice received unilateral hindlimb artery ligation and excision as described previously [12,13]. To understand the function of miR-133a on perfusion recovery, miR-133a was knocked down using cholesterol-conjugated miR-133a antagomir retro-orbitally at a dose of 8 mg/kg body weight in STZ-induced diabetic mice day 1 before HLI; on the other hand, miR-133a was overexpressed in the ischemic limb by using an intramuscular injection of pCMV-miR-133a followed by electroporation day 3 before HLI. Empty vectors were used as negative control.

Blood flow in the ischemic and contralateral nonischemic limbs was measured using laser Doppler perfusion imaging system (LDPI, Perimed Instruments AB, Stockholm, Sweden) at day 0, 3, 7, 14, and 21 after HLI as described previously [14]. Perfusion of the ischemic limb was quantified and presented as percentage to the nonischemic side.

### Immunofluorescence

Mice were killed 21 days after HLI and tibial anterior muscles from the ischemic side were cryo-sectioned in 6 μm increments for immunostainig with anti-CD31 antibody (rat antimouse CD31 Ab; cat: # 550274; BD Pharmingen San Jose, CA), followed by incubation with antirabbit secondary antibody (1:400, Boster, Wuhan, China). Images were acquired on an Olympus IX71 high-magnification microscope. Assessment of capillary densities was analyzed by counting five random high-power (magnification ×100) fields, and was expressed as the number of CD31+ cells per field.

### RNA isolation and quantitative RT-PCR (qPCR)

Total RNA was isolated from muscle tissue or cells by using miRNeasy Mini Kit (Qiagen, Shanghai, China) following the manufacturer’s instructions and then quantified with a spectrophotometer (NanoDrop 1000, Thermo Scientific, Wilmington, Delaware). To detect the level of miRs, miRs were reverse transcribed by Taqman MicroRNA Reverse Transcriptase kit (Applied Biosystems) according to the manufacturer’s recommendations, followed by qPCR using miR133a TaqMan miRNA expression assay (Assay ID: Mm04238263) purchased from Applied Biosystems (Foster City, CA), small nucleolar RNA MBII-202(snoRNA202, Assay ID: 001232) was served as an internal control for miR calculation. The generated *C*_t_ value of each miR was normalized by its respective *C*_t_ value of internal control (Δ*C*_t_). Each miR was then further normalized to the average Δ*C*_t_ value of its control group (ΔΔ*C*_t_). The final fold expression changes were calculated using the equation 2^−ΔΔ*C*_t_^.

### Western blotting

Mouse muscle tissue and cells were homogenized in RIPA lysis buffer containing 1% protease inhibitor cocktail (Sigma–Aldrich, Shanghai, China) as described previously [12]. Equal amounts of protein in homogenate samples were separated in SDS-PAGE (Bio-Rad Laboratories, Hercules, CA, U.S.A) and blotted with primary antibodies and corresponding peroxidase-conjugated secondary antibodies. Primary antibodies against GTP cyclohydrolase 1 (GCH1) (sc-271482), β-actin (sc-47778), and secondary antibodies were obtained from Cell Signaling or Santa Cruz Company. The bound antibody signal was developed using an Immun-Star HRP chemiluminescent kit (Shanghai Tanon Science & Technology, Shanghai, China). Western blot image was obtained by Tanon 5200 Chemiluminescence Imaging System (Shanghai Tanon Science & Technology). Semiquantitative analyses of immunoblots were performed using the ImageJ.

### Biochemical assay

Malondialdehyde (MDA) in mouse ischemic muscle homogenates was measured by using thiobarbituric acid as previously described [15]. The absolute amount of MDA was read from a standard curve prepared from serial dilutions of the primary standard. Activation of NO-sensitive guanylyl cyclase leads to enhanced production of secondary messenger–cyclic guanosine monophosphate (cGMP). We thus assessed tissue cGMP levels in the ischemic muscle to reflect tissue NO bioactivity using an enzyme immunoassay system (R&D Systems, Minneapolis, MN) since NO is not stable after tissue harvest.

### Cell culture and *in vitro* transfection and angiogenesis assay

Human umbilical vein endothelial cells (HUVEC) were isolated from a donor umbilical as described previously [16] and then grew in endothelial cell growth medium (Cell Applications Inc, San Diego, CA) supplemented with 10% fetal bovine serum (FBS, Gibco, Shanghai, China). To mimic the endothelial cells under ischemic condition in HLI models, HUVECs were exposed to hypoxia (2% oxygen, BioSpherix, Lacona, NY) and serum starvation (HSS). The use of human umbilical vein was approved by Guangzhou Medical University Institutional Review Boards. Normal concentration of glucose (5 mM) or high concentration of D-glucose (25 mM) culture medium was used to mimic nondiabetic or hyperglycemia *in vivo*.

*In vitro* transfection of microRNA inhibitor or microRNA mimic was used to knockdown or overexpress miR-133a expression respectively in HUVECs as described previously [17]. Briefly, a reverse transfection protocol using neofx transfection agent (Ambion, Austin, TX) was used to transfect miR-133a inhibitor or miR inhibitor negative control (Life Tech) into HUVECs for 48 h.

*In vitro* angiogenesis assay was performed as previously described under HSS conditions [17]. Briefly, HUVECs transfected with miR-133a inhibitor, mimic, or control were plated at a density of 1 × 10^4^ cells/well on 96-well dishes, which were coated with growth factor-reduced Matrigel (BD Biosciences, MA, U.S.A) and then exposed to HSS conditions for 6 h to assess tube formation. Each condition was done in six wells. The degree of tube formation was determined by measuring the number of loops from each well under 40× magnifications using ImageJ (National Institute of Health, Bethesda, MD). Each experiment was repeated at least in two different batches of HUVECs. DCH1 inhibitor, 2′,4′-diamino-6-hydroxypyrimidine (DAHP, 1 mM); endothelial NO synthase (eNOS) inhibitor, was used for the pathway study.

### Intracellular reactive oxygen species (ROS) and NO assay

Intracellular reactive oxygen species (ROS) production was detected using the cell permeating compound, 2′,7′-dichlorodihydrofluorescein diacetate (DCFH-DA) as described previously [18]. In the presence of ROS, DCFH-DA is hydrolyzed to the fluorescent product. After incubation with 10 µM DCFH-DA for 45 min at 37°C, fluorescence intensity of HUVECs was read at 525 nm emission when excited at 488 nm in a 96-well plate reader. NO level in HUVECs was detected using the fluorescent probe 4-amino-5-methylamino-2′,7′-difluorofluorescein (DAF-FM), which is similar to ROS assay. The DAF fluorescent intensity was recorded by fluorescent reader at the wave of excitation (485 nm) and emission (545 nm). Results were expressed as the percentage of control (cells cultured under normoxia and normal glucose conditions) fluorescence intensity.

### Data analysis and statistics

Statistical analysis was performed with GraphPad Prism software. The unpaired *t* test was used for comparison between two groups and comparisons in experiments with ≥3 groups were performed with one-way ANOVA followed by the Tukey post hoc test. Statistical significance was set at *P*<0.05.

## Results

### Hyperglycemia increased miR-133a under ischemia

**Figure 1:**
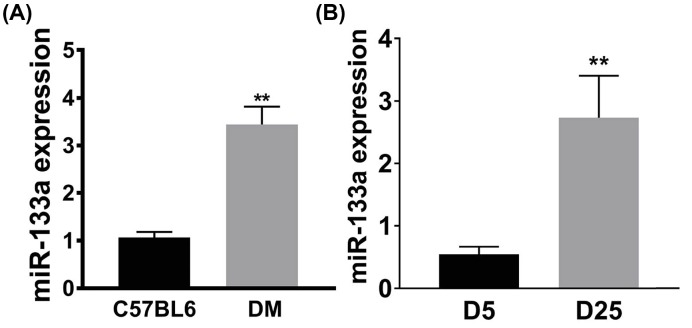
Hyperglycemia induced excessive miR-133a expression in ischemic muscle tissue and endothelial cells under simulated ischemia (**A**) Higher levels of miR-133a protein were expressed in day 7 postischemic gastrocnemius anterior (GA) muscle tissues from mice with type 1 DM compared with day 7 postischemic GA tissues from nondiabetic mice. (**B**) Cultured endothelial cells (HUVEC) exposed to high glucose culture (D25) showed higher level of miR-133a compared with HUVECs exposed to normal glucose (D5) 24 h under hypoxia and serum starvation conditions. Data = mean ± SEM; C56BL6, nondiabetic C57BL6 mice; DM, type 1 DM; **P<0.01.

By using qPCR, we found miR-133a expression level in ischemic mouse hindlimbs was significantly higher from mice with diabetes when compared with nondiabetic mice day 7 after HLI (Figure 1A). Next, we analyzed the expression of miR-133a in HUVECs exposed to normal glucose (5 mM) and hyperglycemia (25 mM) under HSS conditions for 48 h, consistent with what we found in ischemic muscle, miR-133a expression in HUVECs cultured with hyperglycemic culture medium was higher than HUVECs under normal glucose culture (Figure 1B).

### MiR-133a inhibitor improved perfusion recovery and angiogenesis in diabetic mice

**Figure 2:**
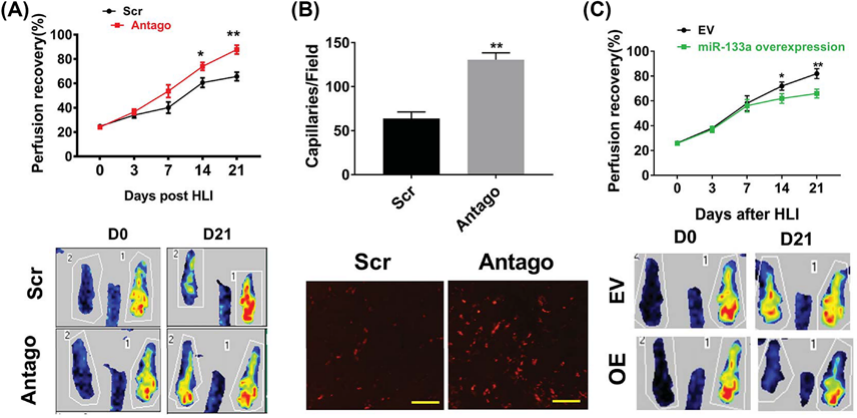
Modulation of miR-133a expression altered perfusion recovery following HLI (**A**) Laser Doppler imaging showed significant increase in perfusion recovery in type 1 diabetic mice transfected with miR-133a antagomir at day 14 and 21 after HLI (*n* = 10/group). (**B**) At day 21 after HLI, ischemic gastrocnemius muscle from mice transfected with miR-133a antagomir showed significantly higher capillary density (CD31, red) when compared with mice transfected with scrambled sequence (*n* = 10/group), bar = 100 µM. (**C**) MiR-133a overexpression in hindlimb decreased perfusion recovery at day 14 and 21 post-HLI in nondiabetic C57BL6 mice. Data = mean ± SEM; Scr indicates scrambled RNA, which serves as a negative control for microRNA transfection, antago indicates mice transfected with miR-133a antagomir; **P<0.01 and *P<0.05.

In the ischemic muscle from STZ-induced diabetic mice, miR-133a level was higher than nondiabetic mice. Thus, we knocked down miR-133a in diabetic mice using antagomir, which showed approximately 80% knockdown in the ischemic muscle day 7 after HLI. Follow-up Laser Doppler imaging showed that miR-133a knockdown improved perfusion recovery compared with those receiving scramble sequence at day 14 and 21 after HLI (Figure 2A). Next, we determined capillary density in the ischemic muscle using immune staining with CD31, mice receiving miR-133a antagomir showed higher capillary density compared with those receiving scramble RNA (81.9 ± 4.3 compared with 44.3 ± 2.7, capillaries/field, *n* = 10/group, *P*<0.01) in the ischemic gastrocnemius muscle day 21 after HLI (Figure 2B).

### Overexpression of miR-133a impaired perfusion recovery

To understand the impact of elevated miR-133a expression on perfusion recovery, we overexpressed miR-133a using pCMV-miR-133a expression plasmid followed by electroporation in the left hindlimb day 3 before HLI in C57BL6 mice. The expression level of miR133a in the ischemic muscle was assessed day 7 after HLI and pCMV-miR-133a plasmid showed approximately 90-fold higher expression of miR-133a compared with mice receiving control plasmid. At day 14 and 21 post-HLI, miR-133a overexpression resulted in impaired perfusion recovery compared with those receiving control plasmid as indicated by LDPI (Figure 2C).

### MiR-133a antagonism decreased ROS, increased GCH1 and cGMP *in vivo*

**Figure 3:**
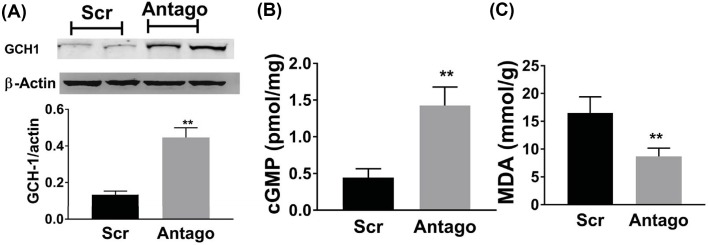
MiR-133a increased cGMP via targeting GCH-1 MiR-133a antagonism increased GTP cyclohydrolase I (GCH-1) (**A**) and cyclic guanine monophosphate (cGMP) (**B**), and decreased malondialdehyde (MDA) (**C**) in the ischemic muscle day 7 after experimental PAD. Data = mean ± SEM; Scr indicates scrambled RNA, which serves as a negative control for microRNA transfection, antago indicates mice transfected with miR-133a antagomir; **P<0.05.

GTP cyclohydrolase 1 (GCH1) has been reported as the rate-limiting enzyme for *de novo* synthesis of tetrahydrobiopterin, which is important for eNOS function. Interestingly, GCH1 is also a target of miR-133a in endothelial cells. Here, we measured GCH1 protein level that was up-regulated in the ischemic muscle when miR-133a was antagonized (Figure 3A). NO is a strong stimulant of angiogenesis and exerts its effects by the stimulation of intracellular cyclic guanine monophosphate (cGMP) production [19], we assessed levels of cGMP in the ischemic muscle and showed 2.6-fold higher (*P*<0.01) cGMP in ischemic hindlimbs from mice receiving miR-133a antagomir when compared with control group, thus providing evidence that miR-133a antagonism increases NO bioactivity in ischemic muscles (Figure 3B). It is known that diabetes impairs vasculature through ROS elevation. Here, we sought to study ischemic muscle tissue ROS through measuring malondialdehyde (MDA), which was widely used to reflect the ROS bioactivity in tissue [20]. MiR-133a knockdown using antagomir significant decreased MDA level in the ischemic muscle day 7 after HLI (Figure 3C).

### MiR-133a modulated angiogenesis through GCH1 *in vitro*

**Figure 4:**
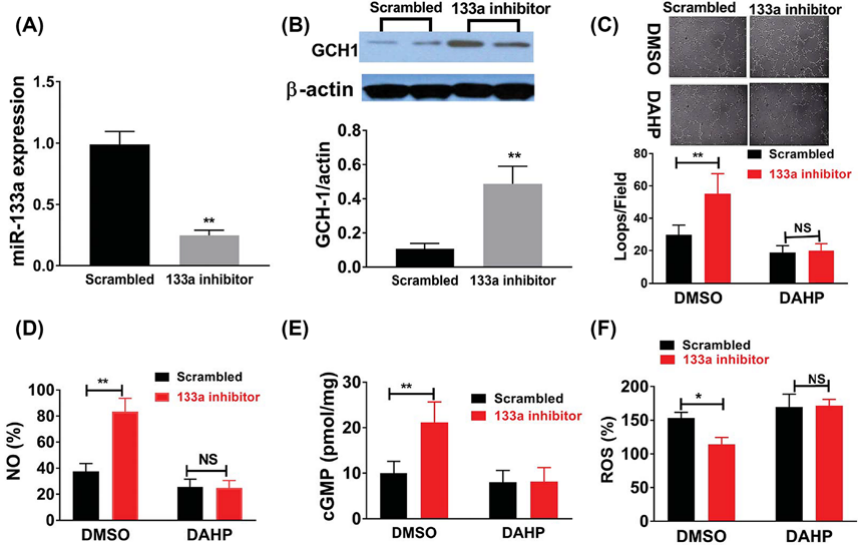
MiR-133a antagonism increased tube formation and cellular NO, cGMP, decreased cellular ROS in cultured HUVECs After transfection with miR-133a inhibitor or scrambled RNA sequences, HUVECs were plated on matrigel with reduced growth factor and incubated for 12 h under high glucose (D25) and HSS condition, miR-133a inhibitor significantly decreased miR-133a level (**A**); showed increased GCH1 protein level (**B**); enhanced tube formation, which was quantified as total length of the cords per visual filed, and total loops per visual field as represented by the bar graph (**C**); increased NO (**D**) and cGMP (**E**) levels, and reduced ROS as indicated by 2′,7′-dichlorodihydrofluorescein diacetate assay (**F**). All the above data are representative of 2–3 separate batch of HUVECs, *n* = 8–12/group. Data = mean ± SEM; NO indicates nitric oxide; Scrambled indicates scrambled RNA sequences; 133a inhibitor indicates miR-133a inhibitor; ROS indicates reactive oxygen specie; cGMP indicates cyclic guanine monophosphate; DMSO indicates dimethyl sulfoxide; DAHP indicates 2′,4′-diamino-6-hydroxypyrimidine, which is a DCH1 inhibitor; NS P>0.05,**P<0.01 and *P<0.05. NS, not significant.

**Figure 5:**
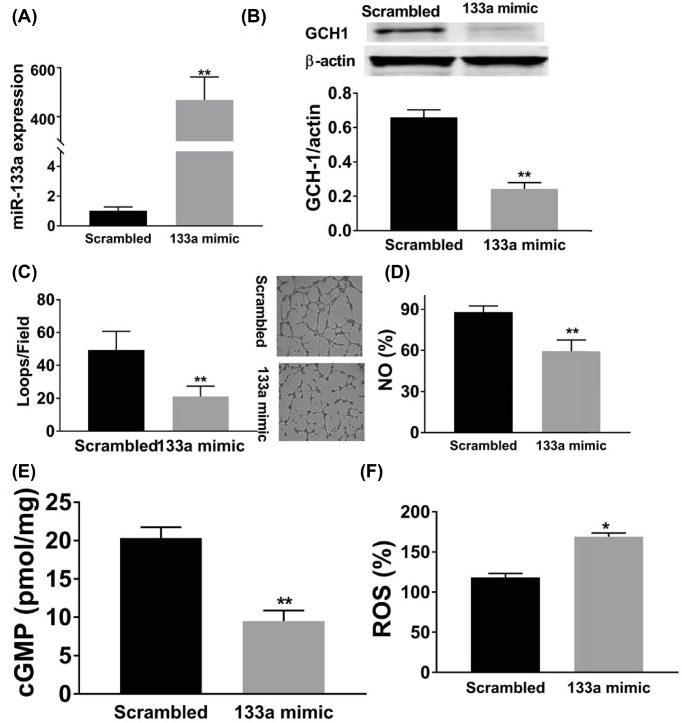
MiR-133a overexpression decreased endothelial cell survival and angiogenesis under simulated ischemia. *In vitro* transfection of miR-133a mimic in HUVECs under normal glucose (D5) increased miR-133a expression (**A**), decreased GCH1 expression (**B**), tube formation (**C**), intracellular NO (**D**) and cGMP (**E**) levels, and increased ROS level (**F**); *n* = 8–12/group; Data = mean ± SEM; NO indicates nitric oxide; Scrambled indicates scrambled RNA sequences; 133a mimic indicates miR-133a mimic; ROS indicates reactive oxygen species; cGMP indicates cyclic guanine monophosphate; **P<0.01 and *P<0.05.

Next, we studied the effects of miR-133a on angiogenesis *in vitro* by using HUVECs cells. Transfection of miR-133a inhibitor significantly decreased intracellular miR-133a level (Figure 4A), and similar to what we found in the ischemic muscle, miR-133a antagonism in HUVECs under hyperglycemia and HSS conditions increased GCH1 protein expression (Figure 4B), tube formation (Figure 4C), NO (Figure 4D) and cGMP (Figure 4E), and decreased ROS level (Figure 4F). In contrary, miR-133a overexpression using microRNA mimic transfection in HUVECs under normal glucose and HSS conditions significantly increased miR-133a expression (Figure 5A), decreased tube formation (Figure 5B), intracellular levels of GCH, NO, cGMP, (Figure 5C–E) and increased intracellular ROS levels (Figure 5F).

## Discussion

To best of our knowledge, this is the first report demonstrates that ischemia-induced miR-133a down-regulation is blunted in diabetes, miR-133a antagonism improves perfusion recovery in diabetic mouse PAD model, and miR-133a overexpression impairs perfusion recovery. We further found that miR-133a targeted GCH1 and increased ROS levels in ischemic muscle tissue and endothelial cells, which resulted in increased NO and cGMP levels in endothelial cells.

It is well known that patients with diabetes are at increased risk of tissue loss and impaired angiogenesis after ischemia [8,21]. Endothelial cell is one of the key cell types that are involved in angiogenesis; however, this cell type is particularly vulnerable to hyperglycemia-induced damages. In present study, miR-133a was found to be higher both in the ischemic tissue and endothelial cells expose to hyperglycemia; in addition, miR-133a knockdown using RNA transfection improved perfusion recovery and angiogenesis in diabetic model. These may suggest that miR-133a is important for diabetes-induced angiogenesis impairment. A number of studies have demonstrated miRs play a key role in regulating the expression of genes that are involved in angiogenesis [17,22,23]. Recent studies showed that diabetes regulates the expression several miRs in endothelial cells, which include miR29a and miR-221 [12,24]. Similar to our findings, miR-29a antagonism reverses diabetes-induced angiogenesis impairment [12]. The mechanisms of impaired angiogenesis caused by diabetes are complicated; discovery of hyperglycemia-induced miRs may provide new clues to understand the effects of diabetes on angiogenesis.

NO is a key molecule for angiogenesis in ischemic disease. Endogenous NO production in endothelial cells is mainly through eNOS, GCH1 is vital for the synthesis of BH4 which coupled with eNOS to generate NO [25,26]. In PAD models with diabetes, excessive miR-133a in ischemic muscle or endothelial cells targeted GCH1 and reduced its protein level, which decreases NO synthesis and increases ROS production in the ischemic muscle tissue or endothelial cells. In PAD patients with diabetes, hyperglycemia-induced generation of reactive oxygen species (ROS) resulting in decreased synthesis and increased degradation of NO [27,28]. Here, in present study, miR133a antagonism decreased ROS and NO level in hyperglycemic endothelial cells and the NO downstream molecule—cGMP.

Taken together, the present study suggests that in PAD models with diabetes, miR-133a up-regulation inhibits NO-cGMP angiogenic pathway through targeting GCH1. As miRs modulation is entering clinical trials [29,30], targeting miR-133a should be an attractive strategy to treat PAD patients, especially when complicated with diabetes.

## Abbreviations

DAF: 4-Amino-5-Methylamino-2′,7′-Difluorofluorescein
eNOS: endothelial nitric oxide synthase
HLI: hindlimb ischemia
HSS: hypoxia and serum starvation
HUVEC: human umbilical vein endothelial cells
MDA: malondialdehyde
NO: nitric oxide
PAD: peripheral arterial disease
RIRA: Radio Immunoprecipitation Assay
ROS: reactive oxygen species
